# Enhancement of nutritional quality and shelf life of fish products (powder & chapatti) via fortifying with orange‐fleshed sweet potato

**DOI:** 10.1002/fsn3.3760

**Published:** 2023-10-16

**Authors:** Habtamu Birhanu Mekonnen, Tewodros Birhanu Aychiluhm

**Affiliations:** ^1^ Department of Chemistry (Food and Sugar Technology Stream), College of Natural and Computational Sciences Arba Minch University Arba Minch Ethiopia

**Keywords:** beta‐carotene, chapatti, fish powder, fortification, sweet potato

## Abstract

Despite its high protein, fat, and mineral contents, fish contains trace amounts of carbohydrates and vitamins, notably vitamin A. The perishable nature of fresh fish makes it challenging to store for a prolonged time, necessitating the use of additives to enhance its shelf life, nutritional, and other quality aspects. Sweet potatoes are the preferred option to blend with fish due to their cost and abundance. This study aims to prepare fortified fish powder and make food products (chapatti) using it. Fish powder and sweet potato powder were prepared by drying them in an oven at 60°C and 45°C, respectively. The two dried samples were then ground and mixed in various ratios, followed by analyzing their nutritional and other parameters using standard methods. Vitamin A and beta‐carotene levels were analyzed using HPLC and UV–Vis spectroscopy techniques, respectively. The findings indicated that the outcomes displayed enhanced nutrition and extended storage capacity. The amount of beta‐carotene (876.12 ± 14.76 to 3182.4 ± 123.1 μg/100 g) and carbohydrates (4.49 ± 1.02 to 52.31 ± 0.21) increased. The packed fortified flour is safe for human consumption for up to 90 days, as per the International Commission for Microbiological Specifications for Foods. The chapatti made from fortified flour was also deemed acceptable by the panelists.

## Graphical abstract

Fish is a highly perishable food item and lacks nutrients like carbohydrates and vitamins. Hence, we developed food products from fish that demonstrated enhanced nutrition and extended shelf life by blending with orange‐fleshed sweet potato. The amount of beta‐carotene (876.12 ± 14.76 to 3182.4 ± 123.1 μg/100 g) and carbohydrates (4.49 ± 1.02 to 52.31 ± 0.21) increased.

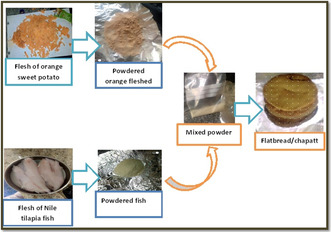

## 1 INTRODUCTION

Both Nile tilapia and sweet potato are locally available. There are many species of Fish in the world. In Ethiopia, 200 species of Fish are available. Among these, Nile tilapia (*Oreochromis niloticus*), the family of *Cichlidae*, contributes more than 50% of the total landings of Fish caught per year (Agumas, 2018). The production potential of Ethiopia has been estimated to be 94,500 tons per year in the past years. Nile tilapia is widely found in Rift Valley lakes in Abaya and Chamo and highland lakes in Tana. It is the most preferred edible fish species in tropical and subtropical freshwater bodies in various African countries (Tesfahun, 2018; Tessema et al., 2019).

Fish is a unique source of both essential macro and micronutrients; it is found in limited amounts in the diet and is highly consumed worldwide (Wake & Geleto, 2019). Fish is a source of protein in high amounts, fatty acids, and minerals (calcium, iron, zinc, and iodine). But Fish has trace amounts of carbohydrates and essential vitamins, especially vitamin A (Abelti, 2017). Tilapia fish are an excellent source of protein and are relatively low in fat. They are rich in niacin, vitamin B12, phosphorus, iron, selenium, and potassium (Jim et al., 2017).

The loss of Fish due to spoilage in the world is estimated to be 10–12 million tons per year, accounting for 10% of total production. Generally, the wastage of Fish through spoilage has been estimated to range from 18% to 30% in developing nations (Mphande & Chama, 2015). It indicates that the habit of preserving and processing Fish into different products before spoilage is poor or not enough. Fish deterioration or spoilage is one of the fishing industry's most significant problems. Thus, processing fish into other products through drying and fortification is very critical to ensure preservation or to increase its shelf life.

Sweet potato (*Ipomea batatas* [L.] *Lam*.) root contains high amounts of carbohydrates and essential minerals like zinc, magnesium, potassium, and calcium. It is an important source of beta‐carotene, vitamin C, and B, but no more protein. In addition, orange‐fleshed sweet potatoes contain phenolic compounds such as hydroxycinnamic acids, which represent the primary phenolic antioxidants that may enhance the nutritional values of the overall food products. Also, it can contribute to the color, flavor, and dietary fiber of processed food products (Sanoussi et al., 2016). Depending on the flesh color, sweet potato is grouped into four types, orange, white, cream, and yellow‐fleshed sweet potatoes. Orange‐fleshed sweet potato (OFSP) also has different varieties; Kulfo (LO‐323), Tulla (CIP 420027), Kero (TIS‐8250), Guntute (AJAC‐I), and Birtukane (saluboro) and are produced in Ethiopia (Gurmu, 2019). Guntute (AJAC‐I) has a good yield of 354 qt/ha and a better maturity period than other varieties (Gurmu, 2019).

According to the Ethiopian Public Health Institute (2019), vitamin A deficiency in Ethiopia is a significant public health problem, especially in preschool children. To reduce these problems, many food producers and researchers develop different food products from various species of Fish and other ingredients. For instance, fish balls produced by fortifying Mrigal carp fish powder with potato (Solanum tuberosum) flour (Chowdhury et al., 2017); pasta from Fish (Pseudophycis Bachus) and wheat (Desai et al., 2018), and “Enbal” from fish meal and sweet potato leaf (Riry et al., 2018). Also, Monteiro et al. (2016) produced pasta from Tilapia (*Oreochromis niloticus*) flour with white wheat flour (*Triticum aestivum* L.) and whole powdered egg. There have been studies done on orange‐fleshed sweet potatoes as a fortifying agent, but there is no study on using orange‐fleshed sweet potato tuber flour to fortify Nile tilapia fish powder to produce fortified fish powder and food products like chapatti (flatbread typically made from wheat flour). Preparing the powder form of fish is advantageous due to its lighter weight, increased portability, and greater stability resulting from low water activity (Shaviklo, 2015). Fortifying the powder with orange‐fleshed sweet potatoes enhances its nutritional value, and the final product can be stored in polyethylene bags to prolong its shelf life. Therefore, the objective of this study is to look into the improvement of the nutritional value and shelf life of food products (powder and flatbread/chapatti) made from fish.

## 2 MATERIALS AND METHODS

### 2.1 Experimental materials

Nile tilapia fish was collected from a local fish processing factory at Chamo, Orange‐fleshed sweet potato tuber local variety (Guntute (AJAC‐I)) at Arba Minch Agricultural Research Institute, and Garlic and table salt were bought from Shecha market, Arba Minch, Gamo Zone, Southern Nations, Nationalities, and Peoples' Region, Ethiopia.

### 2.2 Material preparation

#### 2.2.1 Fish powder

The collected fish samples were washed in potable water several times to remove dirt and debris on the surface and filleted. The process of washing was repeated to remove the microbial load. Then 400 g of minced fish blanched in 10% of brine solution at 100°C for 5 min to retard/inhibit some enzymes or microorganisms susceptible to deterioration or run acidity. The collected garlic was peeled, washed, and cut into small pieces using a knife. Then, 40 g of the sliced (chopped) garlic was added (Begum et al., 2012; Chowdhury et al., 2017) and mixed with the blanched fillet. Then the samples were dried in a hot air oven at 60°C ± 5°C for 15 h milled into powder with the help of a laboratory grinder, and sieved through 0.5 mm screen mesh for the separation of bone (Abraha et al., 2018; Chowdhury et al., 2020; Riry et al., 2018; Santana et al., 2012). Then the fish powder was kept and stored in a polyethylene bag and used for fortification and analysis.

#### 2.2.2 Sweet potato flour preparation

The collected sweet potato roots were washed and cleaned in water to remove soil and other foreign matter. The tuber was peeled with a knife and washed again. Then manually cut into slices (2–4 mm thick) to reduce the sizes and increase their drying surface. The sliced tubers were blanched at 60°C for 5 min in the water bath to inactivate enzymes that may cause a browning reaction. Then the blanched sample was dried at 45°C in the oven for 12 h. The dried slices were milled into a fine powder and passed through a 500 μm sieve using a hammer mill. The fine flour obtained was sealed immediately with aluminum foil until fortification to avoid rehydration and stored in a polyethylene bag (Haruna, 2018; Ngoma et al., 2019; Tortoe et al., 2017).

### 2.3 Nutritional analysis of raw materials

#### 2.3.1 Proximate composition of raw materials

The proximate compositions (moisture content, crude protein, crude fat, ash, crude fiber) of dried fish and orange‐fleshed sweet potato were analyzed according to the standard methods of AOAC (2000, 2006).

#### 2.3.2 Determinations of mineral content in raw material

After the digestion of 1 g of each sample, the absorbance of each mineral was analyzed and determined according to their standard (Bedassa, 2020; Melnikov et al., 2016). Each sample was analyzed in triplicate, and the concentration of each metal was calculated by the following formula as described by Abelti (2017).

$$\text{Metal content}\left(\frac{\mathrm{mg}}{100\;g}\right)=\frac{\left(A\right)\mathrm{VDf}}{10W},$$

where, W, Weight of the sample (g); V, Volume of the extract (mL); A, Concentration (mg/L) of sample solution, and Df is the dilution factor.

#### 2.3.3 Determinations of vitamin A in dried fish

A known quantity of 2.5 g of dried fish was saponified in 40 mL of 95% ethanol and 10 mL of 50% KOH in a round bottom flask. Then, the quantification of vitamin A was done by HPLC (Shimadzu model; made in Japan), with the following conditions: detection: fluorescence detector; excitation: 325 nm; emission: 480 nm; mobile phase: 98:2 Methanol: water; flow rate: 1 mL/min; injection volume: 10 μL; run time: 11 min (Kasozi et al., 2018; Yildirim, 2011).

#### 2.3.4 Determination of Beta‐carotene in orange‐fleshed sweet potato

Since the UV–Vis spectrometric method is simple, sensitive, low cost, and reliable, and commonly used for the determination of β‐carotene content in different food categories (carrot, sweet potato, and carrot and sweet potato and fortified chicken meat nuggets) and was developed and validated (Biswas et al., 2018). So, 2 g of sweet potato flour was measured into a 50 mL conical flask and mixed with 40 mL acetone. The extraction with acetone was continued until the residues became white. Finally, the ethereal extract (petroleum ether phase) was collected and filled with petroleum ether in a 50 mL volumetric flask. The absorbance of the ethereal beta‐carotene extract was read at 450 nm in a UV/VIS spectrophotometer (SPCORD PLUS 50 model, made in Germany) (Chipungu et al., 2017). The ß‐carotene concentration (C in mg/L) was calculated using Lambert–Beer law from measured data of the absorbance as indicated in the following equation according to (Mamo et al., 2014)

$$\text{Amount of beta}-\text{carotene}\;\left(\frac{\mathrm{\mu g}}{100g}\right)=10^{6}\times \frac{\mathrm{MAV}}{\mathrm{\varepsilon L}\times \text{weight of sample}},$$

where, ε is the Molar extinction coefficient for β‐carotene in petroleum ether (138,900 L.mol.‐1.cm‐1), M is the molecular weight of β‐carotene (536.88 g.mol‐1) and L is the path length (generally equal to 1 cm), and V is the volume of extract.

### 2.4 Experimental design for formulations

The prepared samples were blended in different ratios using a mixture design in Minitab version 18 software. The components of fish powder (40‐85 g) and sweet potato flour (15–60 g) were fed into software in the simple lattice (Omosuli et al., 2019). A 5‐point response was customized in Minitab software in DOE mixture design to optimize nutrient composition (proximate, minerals, vitamins A). The optimal ranges of these flour combinations were analyzed for all possible combinations (Table 1).

**TABLE 1:** Experimental design for formulations of Nile Tilapia fish powder with orange‐fleshed sweet potato.

Sample code	Nile tilapia fish powder per 100 g	Orange‐fleshed sweet potato per 100 g
NS51.25	51.25	48.75
NS73.75	73.75	26.25
NS85	85.00	15.00
NS62.5	62.50	37.50
NS40	40.00	60.00

### 2.5 Titrable acidity and pH determinations of fortified powder

#### 2.5.1 pH determination

The pH was measured by weighing 2 g from each sample and homogenized in 20 mL of distilled water. The solution was filtered, and the pH of the filtrate was measured using a pH meter after calibrations using pH 4, pH 7, and pH 10 buffer solution, respectively, by washing the probe with de‐ionized water in each measurement of buffer (Reza et al., 2015).

#### 2.5.2 Titratable acidity (TA)

One gram of fortified sample was measured and mixed with 10 mL of distilled water in an Erlenmeyer flask and shaken well to have uniform homogenization. The filtrate was titrated by using 0.1 M NaOH in the presence of 1 mL of 2% w/v phenolphthalein indicator. The triplicate measurement calculated the percent of titrable acidity using the following formula (Tomovska et al., 2016).

$$\%\text{Acidity}=\frac{\mathrm{Vol}.\text{NaOH}\times 0.64\times \text{conc}.\text{NaOH}}{\text{Weight of sample}}.$$

### 2.6 Microbial analysis of fortified fish powder

Each food product (fortified fish powder) should fulfill the maximum limits of aerobic plate count (APC) and fungi count in colony‐forming unit/g according to the standard. So the aerobic plate count (total viable bacterial counts) and fungi count (yeast and mold counts) were carried out on the fortified samples after fortification (or before sensory analysis) according to (AOAC, 2006) as described by Omosuli et al. (2019). It was checked per 2 weeks and was compared with the food microbiological standards such as the International Commission for Microbiological Specifications for Foods (ICMSF).

#### 2.6.1 Aerobic plate count (APC) analysis

The APC of the composite sample fish powder to sweet potato flour was analyzed. To analyze these samples, buffered peptone water (BPW) and APC were prepared. After sterilization of APC agar, accurately 25 g of each fortified fish sample was measured by digital balance and placed into the different conical flasks, respectively, and dissolved in 225 mL of buffer peptone water (BPW). Six test tubes were prepared and 9 mL of distilled water was added to each test tube. 1 mL of sample solution was added into the first test tubes to prepare 10–1 solutions. From this test tube 1 mL of sample was taken and added to the next test tube to prepare 10–2 solutions and continued in sequential order until to prepare 10–6 sample solutions. Then, 1 mL of solutions was taken in each test tube and added to six different Petri dishes. Then, 20 mL of APC agar was added and shaken slowly. After cooling put it into 30°C in an incubator for 72 h. After 3 days the count, the colony and CFU/g were calculated and compared with standards (Hussain et al., 2016; Omosuli et al., 2019).

$$\mathrm{CFU}/g=\frac{\left(\mathrm{No}.\;\text{of colonies}\times \text{Dilutionfactor}\right)}{\text{voume of sample used}}$$

#### 2.6.2 Total fungal count

Fungal diseases are the result of interactions between pathogens, fish, and the environment. Potato dextrose agar (PDA) is used for the cultivation of fungi. Commercial PDA powder was dissolved into 1 liter of distilled water by measuring 39 g of powder. For complete dissolving, the mixture was boiled followed by shaking with a hand and autoclaved for 15 min at 121°C. Then the total fungus count of fortified fish powder was determined by measuring 10 g of each fortified fish sample and mixed in 100 mL of sterile peptone water which is prepared above. From this mixture, further ten‐fold dilutions were made up to 106, and 0.1 milliliters of each dilution was plated in triplicate on potato dextrose agar (PDA) supplemented with streptomycin to inhibit bacterial growth. Plates were incubated at 25°C and examined daily for 5 days. The mean number of all fungal colonies appearing in the three plates was taken as the average number of colonies per plate for the fortified sample (Al‐Niaeem et al., 2015; Omosuli et al., 2019).

### 2.7 Preparation of chapatti

**FIGURE 1:**
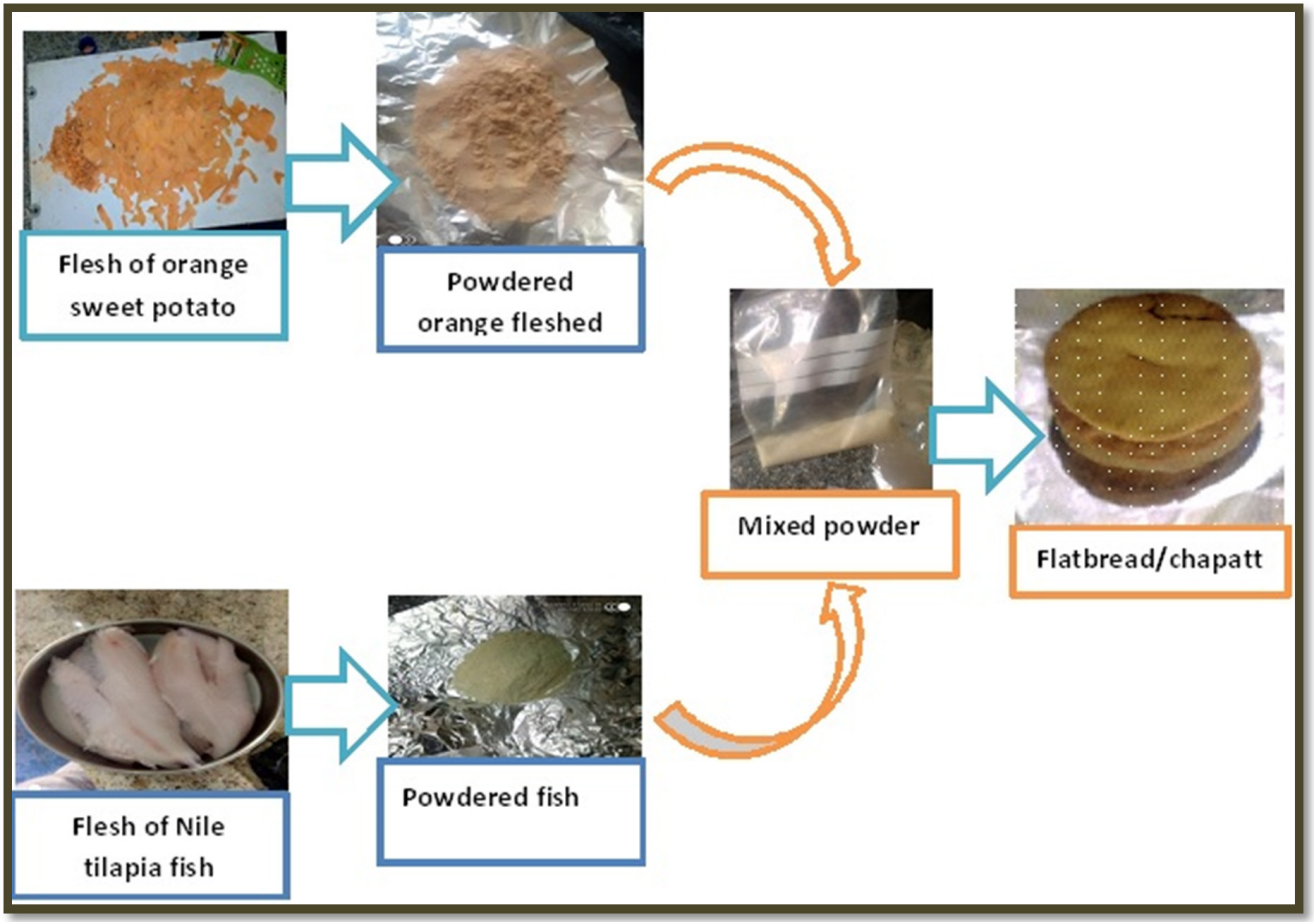
The general work flow for making fortified fish powder and chapatti using fresh orange‐flashed sweet potatoes and fish with different proportions.

Based on Table 1, five different ratios of Nile tilapia fish powder and OFSP were used to make five distinct types of chapatti. A container for mixing dough was filled with 50 g of each blended flour with the ratio, and the dough was then manually kneaded with the addition of 50 mL of water. The dough was flattened/rolled by stick manually, and two tablespoons of sunflower oil were added. Then using aluminum foil, each type of uncooked bread was put in the oven and set the oven temperature at 150°C, which is safe to reduce thermal degradation for 15 min (Afework et al., 2016; De Moura et al., 2015). A hundred percent Nile tilapia fish powder chapatti was prepared as a control. After baking and cooling to room temperature, subjected to sensory and nutritional evaluation (Kadam et al., 2012). Figure 1 shows the overall production process for fortified fish powder and chapatti.

### 2.8 Sensory evaluation and nutritional analysis chapatti

#### 2.8.1 Sensory analysis of chapatti

The sensory characteristics were studied by using the sensory descriptive analysis method. The sensory factors (color/appearance, aroma, taste, texture, and overall acceptability) of each fortified Fish chapatti were analyzed. Then, the chapatti was evaluated using a nine‐point hedonic scale ranging from 1 (dislike extremely) to 9 (like extremely) by 30 Arba‐Minch University Abaya compass untrained panelists by preparing questioners and interviews for the panel (Haripriya & Aparna, 2018; Wartha et al., 2013). They were given coded samples of the prepared bread and invited to assign scores depending on color/appearance, aroma, taste, texture, and overall acceptability using nine‐point numerical measures.

Nine‐point hedonic scale test (including 9 ‐ like immensely, 8 ‐ like very much, 7 ‐ like moderately, 6 ‐ like slightly, 5 ‐ neither like nor a dislike, 4 ‐ dislike slightly, 3 ‐ dislike moderately, 2 ‐ dislike very much, 1 ‐ dislike immensely) was used for evaluating sample acceptability of flatbread (Oguizu et al., 2019).

#### 2.8.2 Proximate composition of fortified fish chapatti

The percentages of fat, fiber, protein, carbohydrate, moisture, and ash content of the flatbread were analyzed according to AOAC (2000, 2006) as described by Jim et al. (2017) and Bibiana et al. (2014) in the above.

#### 2.8.3 Minerals and beta‐carotene in chapatti

The same procedure determined the minerals and beta‐carotene content of each fortified Fish chapatti sample above 2.4.2 and 2.4.4.

### 2.9 Statistical data analysis

All experiments and analyses were performed in triplicate. Data were expressed as mean ± standard deviation (s.d.). All the mean values of the triplicate samples were compared using one‐way analysis of variance (ANOVA) and the regression mixtures of OFSP and Nile tilapia fortified fish flatbread were analyzed using designs of the experiment (DOE) in Minitab version 18.1. Differences were considered statistically significant at *p* ≤ .05.

## 3 RESULT AND DISCUSSIONS

### 3.1 Proximate compositions of raw materials and chapatti

Determining the proximate composition is essential for identifying and acceptance of food products. The proximate compositions (moisture, protein, fiber, fat, ash, and carbohydrate content) of fish, orange‐fleshed sweet potato, and fortified fish flatbread/chapatti are shown in Tables 2, 3, 4, respectively. In general, there is a significant difference (*p* ≤ .05) between the proximate compositions of raw material and bread formulation. The incorporation of orange‐fleshed sweet potato into Niltilapia fish powder reduced the moisture content, ash content, and protein value of the product while increasing fiber, carbohydrate, and beta‐carotene (provitamin A) levels.

**TABLE 2:** Proximate Compositions of garlic‐treated and non‐garlic‐treated Fish.

Parameters	FW	FWO	% CV
Moisture	7.57 ± 0.05^a^	7.97 ± 0.06^b^	1.74
Ash	10.2 ± 0.04^a^	10.5 ± 0.22^a^	2.74
Fiber	0.94 ± 0.04^a^	0.81 ± 0.01^b^	3.79
Protein	71.88 ± 0.13^a^	70.46 ± 0.35^a^	6.75
Fat	7.14 ± 0.16^a^	7.24 ± 0.01^a^	5.27
Carbohydrates	2.27 ± 0.21^b^	3.02 ± 1.36^a^	7.79
Energy (kcal/100 g)	360.87 ± 0.87^a^	359.07 ± 1.93^a^	0.46

*Note*: Values are mean ± standard deviations of triplicate determinations. The mean values that do not share the letter are significantly different. FW is garlic‐treated Fish; FWO is Fish without garlic; % CV is the coefficient of variation.

**TABLE 3:** Proximate compositions of OFSP.

Parameters	Orange‐fleshed sweet potato (OFSP)
Moisture	6.53 ± 0.16
Ash	2.23 ± 0.05
Fiber	3.39 ± 0.03
Protein	3.41 ± 0.16
Fat	1.16 ± 0.17
Carbohydrate	83.1 ± 0.5
Energy (kcal/100 g)	357.17 ± 0.5

**TABLE 4:** Proximate compositions of fortified Fish flatbread/chapatti product in g/100 g.

S.Code	Fiber	Moisture	Ash	Protein	Fat	Carbohydrates	Energy (kcal)
Control	0.78 ± 0.01^f^	13.62 ± 0.35^a^	9.65 ± 0.08^a^	63.36 ± 0.68^a^	8.08 ± 0.12^a^	4.49 ± 1.02^f^	344.16 ± 1.85^c^
NT85	1.06 ± 0.05^e^	10.59 ± 0.42^b^	8.49 ± 0.21^b^	55.82 ± 0.48^b^	7.16 ± 0.14^b^	16.87 ± 0.5^e^	359.23 ± 2.72^ab^
NT73.75	1.45 ± 0.05^d^	9.70 ± 0.13^bc^	8.03 ± 0.36^b^	49.00 ± 0.58^c^	6.20 ± 0.17^c^	25.62 ± 1.07^d^	355.6 ± 0.71^b^
NT62.5	2.07 ± 0.04^c^	9.49 ± 0.39^bc^	6.15 ± 0.04^c^	41.82 ± 0.20^d^	5.97 ± 0.04^c^	34.5 ± 0.52^c^	362.17 ± 1.43^a^
NT51.25	2.61 ± 0.14^b^	8.59 ± 0.41^bc^	5.59 ± 0.04^d^	34.39 ± 0.5^e^	4.5 ± 0.1^d^	44.33 ± 0.69^b^	355.37 ± 1.27^b^
NT40	3.14 ± 0.12^a^	8.44 ± 0.43^c^	4.07 ± 0.03^e^	28.14 ± 0.4^f^	3.9 ± 0.04^e^	52.31 ± 0.21^a^	356.90 ± 2.1^b^
*p*‐value	0.00	0.00	0.029	0.03	0.19	0.051	0.001
% CV	46.76	19.26	28.02	27.4	24.8	55.01	1.68

*Note*: Values are mean ± standard deviations of triplicate determinations. Control (100:0), NT85 (85:15), NT73.75 (73.75:26.25), NT 62.5 (62.5:37.5), NT51.25 (51.25:48.75), and NT (40:60) of Nile tilapia to orange‐fleshed sweet potato. The mean values that do not share the same letters across the column with the same parameter are significantly different.

The moisture content of the fortified bread product exceeded that of each raw material which ranged from 8.440.43 to 13.620.35 g/100 g. Even the control (100% of fish bread) had higher moisture content than its powder as shown in Table 4. This could be because the fortified fish powder has higher water absorption capacity during dough making and baking.

According to Mustapha et al. (2014), the ash content of oven‐dried Nile tilapia fish powder was 13.42 g/100 g. Ash content in the fortified fish flatbread varied from 4.07 ± 0.03 to 9.65 ± 0.08 g/100 g and there were significant differences among them (*p* ≤ .05) (Table 4). Even though the sample's ash content decreased as the OFSP content increased, all fortified fish breads are good sources of minerals.

The National Health and Medical Research Council (NHMRC) (2006) recommended that children and young people between the ages of 2 and 17 should consume 14 and 45 g of protein daily (Ministry of Health, 2012). All of the fortified fish bread samples used in the current study were within the acceptable ranges for protein content, as shown in Table 4. Therefore, fortified flatbread is a healthy source of protein for people.

The fiber content of the fortified fish bread significantly (*p* ≤ .05) increased in all formulations with the addition of the OFSP to dried fish powder and ranged from 1.06 ± 0.05 to 3.14 ± 0.12, as shown in Table 4. The good concentration of crude fiber is an advantage for consumers who eat this fortified product (Neela & Fanta, 2019).

### 3.2 Beta‐carotene and vitamin A content of raw material and fortified chapatti

Vitamin A is found in the form of retinol, which is pre‐formed in animal sources, and in the form of β‐carotene, which is provitamin A in plant sources. Animal foods like fish oil and liver are rich in vitamin A and retinol and are used directly and efficiently by the human body, but poor people cannot afford these expensive foods (Mitra, 2012). The vitamin A content in Nile tilapia fish (muscle) powder is determined using HPLC, while the beta‐carotene content in OFSP flour is determined by UV–vis spectroscopy. The vitamin A content in fish and formulated flat bread/chapatti and the beta‐carotene value in orange‐fleshed sweet potato are presented in Tables 5 and 6, respectively.

**TABLE 5:** Vitamin A and beta‐carotene content (μg/100 g) of dried Fish with garlic (FW) and without garlic (FO) and OFSP and flatbread samples.

Sample code	Beta‐carotene	Vitamin A
FW (control)	–	37.16 ± 0.21^b^
FWO	–	37.24 ± 0.20^b^
OFSP	6194 ± 18^a^	1034.4 ± 29.4^a^

*Note*: OFSP is orange‐fleshed sweet potato; FW is Fish with garlic; FWO is Fish without garlic. 1 μg of beta‐carotene is equal to 0.167 μg of retinol equivalent (RE) (WHO/FAO, 2004). The mean values that do not share the same letters across the column with the same parameter are significantly different.

**TABLE 6:** Beta‐carotene content and Vitamin A in formulated flatbread sample.

S.Code	Beta‐carotene in (μg/100 g)	Vitamin A in μg/100 g
Control	–	37.16 ± 0.21^f^
NT85	876.12 ± 14.76^e^	146.31 ± 2.47^e^
NT73.75	1497.8 ± 25.6^d^	250.13 ± 4.27^d^
NT62.5	1990.6 ± 58.8^c^	332.43 ± 9.82^c^
NT51.25	2383.6 ± 56.6^b^	398.05 ± 9.46^b^
NT40	3182.4 ± 123.1^a^	531.5 ± 20.6^a^
*p*‐value	0.00	0.00
% CV	64.02	64.02

*Note*: Control (100:0), NT85 (85:15), NT73.75 (73.75:26.25), NT62.5 (62.5:37.5), NT51.25 (51.25:48.75), and NT (40:60) of Nile tilapia to orange‐fleshed sweet potato. The mean values that do not share the same letters across the column with the same parameter are significantly different.

**FIGURE 2:**
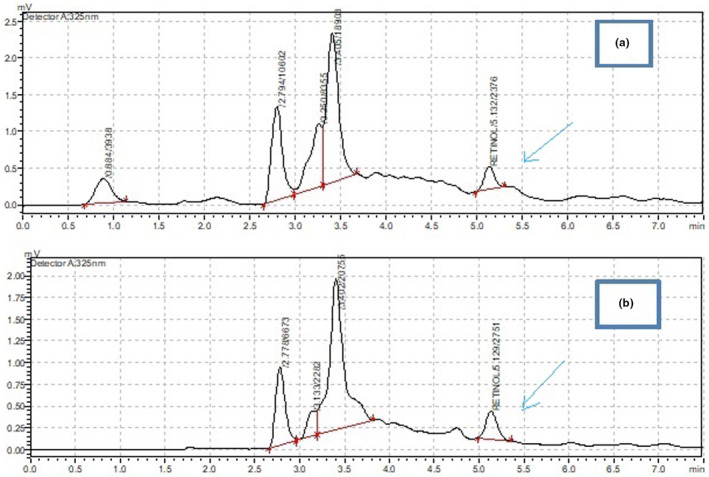
The chromatogram demonstrating the analysis of retinol in fish samples. The peak areas with retention time 5–5.5 min (38,035 mv‐min) (a) and (37,985 mv‐min) (b) corresponds with the amount of retinol (vitamin A) for sample solution of fish with garlic and fish without garlic, respectively. Standard retinol is used for HPLC analysis at 325 nm.

**FIGURE 3:**
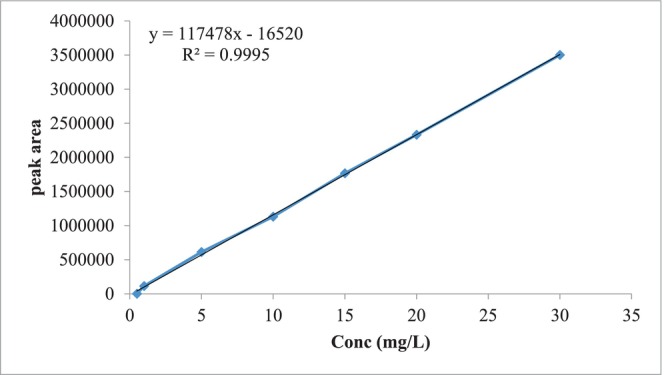
Calibration curve of retinol used for vitamin A quantification in sample solution using HPLC at 325 nm. It relates peak area with concentrations of standard retinol in μg/mL (0.5–30).

In the determinations of vitamin A, the saponification reaction was performed to break the ester bonds, followed by the extraction of the samples before the injection into the HPLC systems. In the HPLC detector at 325 nm, the chromatograms for 0.5, 1, 5, 10, 15, 20, and 30 μg/mL standard retinol solutions were obtained. In the same retention time at 5–5.5 min by different peak areas, the chromatograms of the retinol in the sample solution (garlic‐treated fish and non‐garlic‐treated fish powder) were displayed in the detectors (Figure 2a,b) and Figure 3 for standard retinol.

The concentration of vitamin A was determined in the calibration curve that was obtained by plotting the area of the standard retinol peaks and the retinol concentration. Thus, the concentration of retinol in the samples was determined by applying the linear regression equation with a correlation coefficient *R* ^2^ = 0.9995 (Figure 3).

In this study, the vitamin A content in Nile tilapia fish muscle powder was 37.24 ± 0.2 μg/100 g and 37.16 ± 0.21 μg/100 g of retinol in non‐garlic treated and garlic‐treated, respectively. This is a low concentration relative to the vitamin A requirement for the human body.

However, the beta‐carotene content in OFSP flour had 6194 ± 18 μg/100 g of β‐carotene and no beta‐carotene in fish powder (Table 5). According to the WHO/FAO (2004) expression, the vitamin A activity of carotenoids provitamin A in diets, 1 μg of beta‐carotene is equal to 0.167 μg of retinol equivalent (RE) or 1 μg retinol equivalent (RE) is equal to 6 μg of beta‐carotene (Ministry of Health, 2012; Turner & Burri, 2001). Therefore, 6194 ± 176 μg/100 g of β‐carotene can provide 1034.4 ± 29.4 μg/100 g of RE. This can meet the vitamin A needs in the diet since the recommended safe level of vitamin A in different age groups ranges from 300 to 900 μg/100 g (Ministry of Health, 2012). So after the conversions of beta‐carotene into vitamin A, the vitamin A content in OFSP was higher than Nile tilapia fish powder.

**FIGURE 4:**
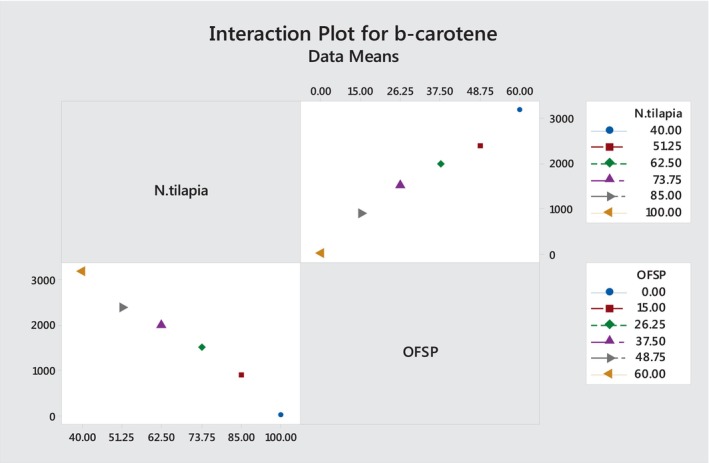
The interaction plot illustrates how the ratios of OFSP and fish powder have an impact on the amount of beta‐carotene in chapatti, which acts as the response parameter. It is generated using Minitab version 18 software. The beta‐carotene content of the chapatti increased as the percentage of OFSP was increased (0–60%, right top corner); however, it dropped when the percentage of Nile tilapia fish powder was increased (40%–100%, left bottom corner).

Figure 4 shows the interaction plots of beta‐carotene in blending OFSP into Nile tilapia fish powder in a fortified chapatti. When OFSP was increased in Nile tilapia fish powder, the beta‐carotene content was significantly (*p* ≤ .05) increased in fortified Fish flatbread. As shown in Table 5, incorporating 26.25% of OFSP gave 1497.8 ± 25.6 μg/100 g of beta‐carotene and 250.13 ± 4.27 μg/100 g of vitamin A. This result slightly agreed with the reported beta‐carotene content of biscuits (prepared with 30% OFSP and 70% wheat flour) in different baking temperatures and times ranging from 286 μg/100 g‐601 μg/100 g (Afework et al., 2016). Also, Nzamwita et al. (2017) reported 597 μg RAE/100 g vitamin A activity in OFSP‐wheat (30:70) composite bread. In the present study, 531.5 ± 20.6 μg/100 g of vitamin A activity was obtained in maximum additions of OFSP in a fortified fish chapatti. Thus, if 2–8 years old children eat the fortified fish chapatti, which is prepared by incorporating 15–51.25% of OFSP into Nile tilapia powder, they can get 48.77–99.5% of the daily recommended dietary intake of vitamin A and if 9 years of old children and adults eat 51.25–60%, OFSP incorporated fortified fish bread can get 66.33–75.92% from the daily recommended intakes of vitamin A (Tables 5 and 6). Therefore, this result suggests that OFSP flour‐enriched chapatti has added advantages nutritionally since it provides vitamin A, indicating the vital role of OFSP in alleviating VAD (Kurabachew, 2015).

### 3.3 Analysis of pH and titrable acidity of fortified flour

The freshness of the fish and fish products can be influenced by pH value because the loss of their freshness can affect bacterial growth. Thus, pH value is an essential index of the quality of the dried fish or the fortified product (Jahan et al., 2019; Rasul et al., 2018). The lowest pH values of dried fish and fish products may inhibit microbial growth and extend the shelf life by reducing the activity of the endogenous enzyme (Rasul et al., 2018). Fish products are acceptable up to a pH of 6.8 but are considered to be spoiled above a pH of 7.0 (Amponsah et al., 2018). So in this study, the pH of both raw materials and each fortified fish powder oscillates between 5.61 ± 0.05–6.53 ± 0.11. This indicates the freshness of fish after oven drying and fortification. No significant difference in the means of the sample pH.

Titrable acidity (TA) refers to the sample's total acidity and is a better indicator of the microbiological stability of certain foods. According to (ICMSF, 1986), titrable acidity is a measure of the quantity of standard alkali (usually 0.1 M NaOH) required for neutralizing acid solution (Busta et al., 2003). In this study, the titrable acidity in all samples showed no significant difference (*p* ≥ .05) between their mean and ranged between 0.04 ± 0.002%–0.05 ± 0.003% see Table 7.

**TABLE 7:** Results of pH and titrable acidity of Nile tilapia fish powder: OFSP flour mixture.

Sample code	pH	% acidity
FW	6.53 ± 0.035	0.05 ± 0.002
FWO	6.23 ± 0.08	0.05 ± 0.003
NT40	6.03 ± 0.08	0.047 ± 0.002
NT51.25	5.98 ± 0.08	0.04 ± 0.003
NT62.5	6.28 ± 0.03	0.04 ± 0.003
NT73.75	6.41 ± 0.12	0.038 ± 0.003
NT85	6.53 ± 0.11	0.036 ± 0.002
OFSP	5.62 ± 0.05	0.034 ± 0.002

### 3.4 Mineral content of raw material and chapatti

In this study, four macrominerals (Na, K, Ca, and Mg), four microessential minerals (Cu, Fe, Mn, and Zn), and two non‐essential (toxic) metals (Cd and Cr, but Cr are not always toxic) were determined in Nile tilapia fish powder, OFSP flour, and chapatti by using AAS. The results are presented in Tables 8 and 9.

**TABLE 8:** Results minerals (macro and microelements) in mg/100 g in raw materials.

Macrominerals				
S. code	Mg	Ca	K	Na
FWO	76.34 ± 0.56^b^	249.5 ± 1.26^a^	297.29 ± 4.94^a^	199.02 ± 0.46^a^
FW	75.77 ± 1.72^b^	244.47 ± 1.65^a^	297.91 ± 4.61^a^	200.08 ± 0.79^a^
OFSP	151.11 ± 0.8^a^	208.95 ± 8.98^b^	135.45 ± 1.43^b^	118.26 ± 0.46^b^
% CV	37.5	8.4	33.32	23.57

Micro/trace elements						
S. code	Fe	Mn	Zn	Cu	Cr	Cd
FW	18.82 ± 0.12^a^	0.34 ± 0.01^a^	3.48 ± 0.12^a^	0.84 ± 0.04^a^	0.35 ± 0.01^a^	0.1 ± 0.009^a^
FWO	17.9 ± 0.07^a^	0.36 ± 0.01^b^	3.54 ± 0.12^a^	0.86 ± 0.02^a^	0.32 ± 0.02^a^	0.12 ± 0.009^a^
OFSP	3.01 ± 0.03^b^	0.25 ± 0.01^c^	0.49 ± 0.03^b^	0.16 ± 0.02^b^	Nd	Nd
% CV	58.01	16.96	60.45	56.31	75.34	77.64

*Note*: The mean values that do not share the same letters across the column or with the same parameter are significantly different.

**TABLE 9:** Results of mineral contents (macro and trace metals) of fortified fish flatbread product essential in mg/100 g.

Macrominerals				
S. code	Mg	Ca	K	Na
Control	76.41 ± 0.69^f^	230.33 ± 1.77^a^	282.04 ± 7.6^a^	183.9 ± 5.07^a^
NT85	86.27 ± 0.11^e^	226.99 ± 1.77^a^	257.45 ± 1.94^b^	176.31 ± 1.2^b^
NT73.75	95.79 ± 0.23^d^	212.85 ± 0.84^b^	239.09 ± 1.43^c^	166.32 ± 1.58^c^
NT62.5	103.26 ± 0.11^c^	204.94 ± 0.67^c^	225.39 ± 0.9^d^	156.33 ± 1.2^d^
NT51.25	115.65 ± 0.13^b^	198.9 ± 0.33^d^	213.25 ± 0.93^e^	153.7 ± 0.8^de^
NT40	108.28 ± 0.47^a^	194.59 ± 0.33^e^	195.21 ± 1.43^f^	148.97 ± 2.09^e^
*p*‐value	0.002	0.5	0.09	0.08
% CV	13.97	6.55	12.52	7.96

Micro/trace elements						
S. code	Fe	Mn	Zn	Cu	Cr	Cd
Control	18.3 ± 0.31^a^	0.32 ± 0.01^a^	3.31 ± 0.03^a^	0.73 ± 0.04^a^	0.32 ± 0.02^a^	0.13 ± 0.02
NT85	16.01 ± 0.31^b^	0.31 ± 0.02^ab^	3.05 ± 0.08^b^	0.61 ± 0.04^b^	0.05 ± 0.02^b^	Nd
NT73.75	13.48 ± 0.16^c^	0.28 ± 0.01^abc^	2.43 ± 0.03^c^	0.43 ± 0.06^c^	0.03 ± 0.01^c^	Nd
NT62.5	11.93 ± 0.09^d^	0.29 ± 0.01^abc^	2.26 ± 0.03^d^	0.3 ± 0.02^cd^	Nd	Nd
NT51.25	10.03 ± 0.03^e^	0.27 ± 0.01^bc^	1.72 ± 0.06^e^	0.27 ± 0.04^d^	Nd^d^	Nd
NT40	9.11 ± 0.03^f^	0.26 ± 0.01^c^	1.48 ± 0.03^f^	0.26 ± 0.04^d^	Nd^d^	Nd
*p*‐value	0.003	0.7	0.66	0.006	0.1	Nd
%CV	25.25	8.59	28.44	42.03	12.91	Nd

*Note*: The mean values that do not share the same letters across the column or with the same parameter are significantly different.

#### 3.4.1 Macro and microelements of raw materials

As shown in Table 8, different macrominerals have other contents in Nile tilapia fish powder and OFSP flour. In fish powder, K > Ca > Na > Mg, and in OFSP, Ca > Mg > K > Na. The Mg content of OFSP was greater and significantly different from Nile tilapia fish powder which was 151.11 ± 0.8 mg/100 g, whereas Nile tilapia was 75.77 ± 1.72 mg/100 g. But fish powder's K, Ca, and Na content were greater than OFSP flour. The Ca, K, and Na content of Nile tilapia fish powder was 244.47 ± 1.65, 297.29 ± 4.94, and 200.08 ± 0.79 mg/100 g, respectively. Bayissa et al. (2021) reported that some macrominerals of Nile tilapia fish filet in dry bases in three different Ethiopia lakes that are 220–240 mg/100 g for Ca, 100–300 mg/kg for Na and 80–140 mg/100 g for Mg which is slightly agreed with the present result. Also, the Na and K content in Nile tilapia powder is agreed with 153.204 ± 2.99–207.182 ± 4.69 mg/100 g and 140.21 ± 8.00–186.16 mg/100 g in dry bases, respectively, as reported by Tsegay et al. (2016).

Iron (Fe) is an essential mineral in trace elements, and its deficiency is the cause of anemia (Azaman et al., 2015). In this study, the iron content of OFSP and Nile tilapia fish powder have significant differences between their mean (*p* ≤ .05), and their result was 3.01 ± 0.03 and 18.82 ± 0.12 mg/100 g, respectively. The iron content of Nile tilapia fish powder was lower than 22.8–24.9 mg/100 g, which was reported by Bayissa et al. (2021). Also, Nicanuru et al. (2015) reported that the iron content of dried OFSP was 1.19–5.1 mg/100 g, which agreed with the present result (Bayissa et al., 2021; Nicanuru et al. (2015)).

The concentration of Cu determined in the Nile tilapia fish powder and OFSP flour was 0.84 ± 0.038 and 0.16 ± 0.02 mg/100 g, respectively. This concentration of Cu in Nile tilapia was above 0.16–0.5 mg/100 g, as reported by Bayissa et al. (2021), and it was lower than 1.08 mg/100 g reported by Ejike and Liman (2017) in the same species and tissues of fish in dry bases.

Mn and Zn are essential trace elements that have different functions or roles in the human body. Also, they are toxic when above their limit, and their deficiencies lead to causes of various diseases (Azaman et al., 2015). In the present study, the concentrations of Mn and Zn in the OFSP and Nile tilapia fish powder were 0.25 ± 0.1 and 0.49 ± 0.03 and 0.34 ± 0.01 and 3.48 ± 0.13 mg/100 g, respectively. The result of Mn and Zn in Nile tilapia dried fish muscle was agreed with 0.23 mg/100 g as reported by Reda and Ayu (2016) and 2.2 ± 2.31–3.47 ± 1.06 mg/100 g (Tsegay & Natarajan, 2016), respectively.

Cr is toxic when it is in the form of Cr (VI), and Cr (III) is essential, but Cd is highly toxic even at a trace level. In this study, the Cr and Cd content of Nile tilapia fish powder were 0.35 ± 0.1 and 0.1 ± 0.009 mg/100 g, respectively, while in OFSP flour, both Cr and Cd were not detected.

#### 3.4.2 Micro and microelements (trace metals) of fortified fish chapatti

The amount of Mg, Ca, K, and Na content of the composite chapatti is presented in Table 9 below. When incorporating OFSP flour from Nile tilapia fish powder, the Mg content was significantly increased while the Ca, K, and Na significantly decreased. This shows that Nile tilapia fish powder is more macromineral‐rich than OFSP. In trace metal, the iron content of the composite flour was significantly reduced with the incorporation of OFSP and ranged between 9.11 ± 0.03–16.01 ± 0.31 mg/100 g. These concentrations of Fe agreed with WHO recommended levels of Fe in the food that is 10–30 mg/100 g limit set by WHO (1982) as described by (Ofori et al., 2016).

In the determinations of Cu, the concentrations of Cu were significantly decreased with *p* ≤ .05 as OFSP was increased and lay between 0.26 ± 0.04–0.61 ± 0.04 in mg/100 g. According to WHO, as described by Ofori et al. (2016), the limit of Cu in food is 0.4 mg/100 g, and thus most of the composite flatbread is within the recommended levels of WHO.

The Mn and Zn content ranged between 0.26 ± 0.01–0.31 ± 0.02 and 1.48 ± 0.03–3.05 ± 0.08 mg/100 g, respectively. Both metals were decreased significantly (*p* ≤ .05) as OFSP incorporation was increased. Based on WHO (2003), the daily recommended allowance of zinc content for adults, including pregnant/lactating women and children, was 3–10 mg/100 g (Laelago et al., 2015; Ministry of Health, 2012). The recommended daily allowance (RDA) of Mn for adult men and women is 2.3 and 1.8 mg/day, respectively (Ho et al., 2012). Thus, the fortified fish chapatti was prepared by one of the composite ratios per 100 g; one can get the daily allowance of Zn and at least 0.26 mg/100 g of Mn.

The Cr content ranged from 0.05 ± 0.02 and 0.03 ± 0.01 for NT85 and NT73.75, and the Cd content was not detected throughout the composite flatbread. The others were non‐detectable as the amount of OFSP increased (Table 9). According to FAO/WHO, the recommended maximum permissible value of Cr is 0.015 mg/100 g. The maximum permitted limit of Cd is 0.05 mg/100 g (Gure et al., 2019).

### 3.5 Shelf life analysis of the fortified powder

#### 3.5.1 Total plate count (TPC) and Total fungus (TF)

Both aerobic plate count and total fungus are the main microbiological criteria that have been used in food production and the food regulatory context for many years. The presence of these pathogenic loads in dried fish is acquiring importance in view of the safety and quality of food (Ahmed et al., 2020; Logesh et al., 2012). In the composite powder product, both total plate count (TPC) and total fungus (TF) were analyzed and presented in Table 10, since they are good indicators of quality or the expected shelf life of the product (Abelti, 2013).

**TABLE 10:** Aerobic plate count and total fungus of fortified powder during 90 days of storage.

S.Code	0DAY	15DAY	30DAY	45DAY	60DAY	90DAY
Aerobic plate count (APC) in cfu/g during 90 days of storage						
F40	5.2 × 10^4^ ± 15^aF^	6.6 × 10^4^ ± 20^Ae^	7.6 × 10^4^ ± 15^aD^	8.7 × 10^4^ ± 17^aC^	1.1 × 10^5^ ± 25^Ab^	1.3 × 10^5^ ± 2.5^aA^
F51.25	4.8 × 10^4^ ± 10^bE^	6.2 × 10^4^ ± 15^abD^	6.6 × 10^4^ ± 26^bD^	8.0 × 10^4^ ± 10^Bc^	1.0 × 10^5^ ± 2.5^abB^	1.2 × 10^5^ ± 2.0^abA^
F62.5	TFTC^cF^	5.6 × 10^4^ ± 20^bcE^	6.2 × 10^4^ ± 10^bcD^	7.6 × 10^4^ ± 10^cC^	9.7 × 10^4^ ± 2.5^bB^	1.1 × 10^5^ ± 4.0^bA^
F73.75	TFTC^cF^	5.2 × 10^4^ ± 10^cE^	6.0 × 10^4^ ± 10^cD^	7.1 × 10^4^ ± 10^dC^	8.4 × 10^4^ ± 2.1^cB^	9.8 × 10^4^ ± 3.6^cA^
F85	TFTC^cF^	4.5 × 10^4^ ± 30^dE^	5.8 × 10^4^ ± 20^cD^	6.5 × 10^4^ ± 11^eC^	7.7 × 10^4^ ± 3.1^dB^	9.6 × 10^4^ ± 3.2^cA^
Total fungus count(TFC) in cfu/g during 90 days of storage						
F40	2.3 × 10^4^ ± 10^aD^	2.8 × 10^4^ ± 1.5^aC^	5.2 × 10^4^ ± 15.3^aB^	5.1 × 10^4^ ± 1.5^aB^	5.5 × 10^4^ ± 10^aB^	8.4 × 10^4^ ± 3.2^aA^
F51.25	2.0 × 10^4^ ± 57^bE^	2.4 × 10^4^ ± 2.1^bE^	3.7 × 10^4^ ± 10^bD^	4.5 × 10^4^ ± 5.7^bC^	5.3 × 10^4^ ± 3.1^aB^	6.3 × 10^4^ ± 3.1^bA^
F62.5	TFTC^cE^	TFTC^cE^	2.9 × 10^4^ ± 2.5^cD^	3.7 × 10^4^ ± 15^cC^	4.4 × 10^4^ ± 2.1^bB^	5.1 × 10^4^ ± 4.6^cA^
F73.75	TFTC^cD^	TFTC^cD^	2.6 × 10^4^ ± 10^cC^	2.8 × 10^4^ ± 2.1^dC^	3.8 × 10^4^ ± 2.1^cB^	4.4 × 10^4^ ± 3.9^cdA^
F85	TFTC^cD^	TFTC^cD^	TFTC^Dd^	2.3 × 10^4^ ± 2.5^eC^	3.2 × 10^4^ ± 15^dB^	4.2 × 10^4^ ± 1.5^dA^

*Note*: a, b, and c: the means that do not share the letter across a column show significant differences within the sample in the same days of storage. A, B, C, D, E, and F: the mean that do not share the letter in a raw shows significant difference within the different days of storage in the same sample.

The International Commission for Microbiological Specifications for Foods (ICMSF, 1986) which is described by Hussain et al. (2016) briefly discussed the quality levels of food in TPC amounts. According to this, the quality levels based on the plate counts unit less than 5 × 10^5^ cfu/g is considered as good quality while the plate count between 5 × 10^5^ and 10^7^ cfu/g is marginally accepted quality and plate count at or above 10^7^ cfu/g is considered as unacceptable for human consumption (Hailemichael & Gutema, 2021; Hussain et al., 2016). In this study, the highest TPC value of the product was not exceeding 1.3 × 10^5^ cfu/g during 90 days of storage at room temperature. This is regarded as all fortified fish powders microbiologically have good quality and are safe for human consumption until 90 days of storage.

However, not all sample products have the same microbial load starting in 0 days to 90 days of storage. This may be the different proportionalities of garlic‐treated fish powder and OFSP flour ratio; as the amount of garlic‐treated fish powder reduced the TPC value was increased across the sample on the same days of storage (Table 10). Also in total fungus count, there was a slight difference with *p* ≤ .05 in both the sample differences in columns and storage time differences in rows. But the highest total fungus count was not exceeding 54 × 10^5^ cfu/g which is 8.4 × 10^4^ cfu/g in all fortified samples throughout 90 days of storage and it is safe for human consumption.

### 3.6 Sensory evaluations of chapatti

Many food products' quality can only be properly assessed by evaluation of their sensory characteristics. The primary measure and a good indicator in examining sensory aspects of food is its flavor or mouth feel. Without a pleasing flavor or mouthfeel, the food is unsatisfactory, regardless of whether the product satisfies the basic nutritional requirement. These sensory qualities included color/appearance, taste, aroma, texture, and overall acceptability. The findings of the sensory evaluation of the fortified fish flatbread with various levels of OFSP added are shown in Table 11. Adding OFSP to dried fish powder mostly alters the flatbread's flavor and aroma. This might be because OFSP has sweet, highly organoleptic qualities.

**TABLE 11:** Sensory quality evaluation of flatbread prepared from composite flours.

Sensory parameters of fortified fish flatbread in different ratios of Nile tilapia to OFSP					
S.Code	Color/appearance	Aroma/smell	Taste	Texture	Overall acceptability
Control	6.5 ± 0.57^b^	6.75 ± 0.96^b^	7.0 ± 0.82^c^	7.5 ± 0.57^a^	7.0 ± 0.82^b^
NT85	7.75 ± 0.96^ab^	7.0 ± 0.82^b^	7.25 ± 0.5^bc^	7.75 ± 0.96^a^	7.75 ± 0.96^ab^
NT73.75	8.5 ± 0.57^a^	8.5 ± 0.58^ab^	7.75 ± 0.57^abc^	8.75 ± 0.5^a^	8.5 ± 0.58^a^
NT62.5	8.25 ± 0.5^a^	8.5 ± 0.58^a^	8.0 ± 0.82^abc^	7.75 ± 0.95^a^	8.75 ± 0.5^ab^
NT51.25	8.0 ± 0.82^ab^	8.75 ± 0.5^a^	8.5 ± 0.58^ab^	7.25 ± 0.5^a^	8.23 ± 0.5^ab^
NT40	7.5 ± 0.58^ab^	8.25 ± 0.96^a^	8.75 ± 0.5^a^	7.5 ± 0.58^a^	7.77 ± 0.5^ab^

*Note*: Values are expressed as mean ± standard deviations. Control (100:0), NT85 (85:15), NT73.75 (73.75:26.25), NT62.5 (62.5:37.5), NT51.25 (51.25:48.75), and NT40 (40:60) of Nile tilapia to orange‐fleshed sweet potato. The mean values that do not share the same letters across the column or with the same parameter are significantly different.

Overall acceptability was determined based on quality scores obtained from evaluating the flatbread's color, texture, taste, aroma, and flavor. The mean score of the overall acceptability of flatbread revealed that 37.5% of OFSP‐supplemented chapatti achieved the maximum score (8.75 ± 0.5). However, the members accepted all the prepared chapatti/flatbread samples for color, texture, taste (mouth feel), and flavor (aroma). Therefore, all chapatti samples were generally acceptable to the untrained panelist.

### 3.7 Response optimization using nutritional values of fortified chapatti

The most important ratios influencing the optimum yield were to be discovered through optimization. The method of optimization involves identifying the best values. The best yield in proximate, minerals, and vitamin A can be obtained in DOE by optimizing the response parameters mixture design, which is a dependable technique. Minitab version 18 was used to choose the mixture components ratio flatbread with the best response value for nutritional values (protein, carbohydrate, beta‐carotene, vitamin A, iron, and magnesium).

The ratio of 61.82:38.18 was the best response for nutritional value, according to nutritional values. This means that when using Minitab software, the composition ratio of Nile tilapia to OFSP yields the optimum nutritional values (protein, carbohydrate, beta‐carotene, vitamin A, iron, and magnesium) at 61.82:38.18 (this is the theoretical result) with composite desirability 0.9008 in using Minitab software (it is the theoretical result).

The fortified chapatti with NT6 (62.5:37.5 Nile tilapia to OFSP) had the highest acceptance rate, as demonstrated in Table 12. Specifically, the panelist chose the flatbread/chapatti prepared with a ratio of 62.5:37.5 (based on sensory testing). This ratio is similar to 61.82:38.18, which provides an optimum yield in terms of nutritional content. Additionally, the nutritional values of the mixture of 62.5:37.5 agree with the theoretical ratio of 61.82:38.18. Therefore, both the nutritional value and the sensory assessment result are superior in the ratios of 61.82:38.18 and 62.5:37.5.

**TABLE 12:** Optimization of Composite chapatti in different nutritional values.

Global solution for composite ratio	Predicted response parameters	Predicted response values	Desirability
N. tilapia = 61.8182 OFSP = 38.1818	Protein	41.61	0.884685
	Carbohydrates	35.91	0.659203
	Beta‐carotene	2026.44	0.909640
	Vitamin A	333.37	0.972007
	Fe	11.80	0.84704
	Mg	102.88	0.767692
	Composite desirability		0.9008

## 4 CONCLUSION

The current investigation successfully produced fortified fish powder and flatbread using Nile tilapia fish and OFSP flours prepared through oven drying. The findings indicated that the overall microbial load and fungi of all packaged composite flour remained within the acceptable limit, ensuring safe consumption for up to 90 days. By incorporating 37.5% OFSP into Nile tilapia fish powder, the fortified fish flatbread products exhibited superior sensory qualities and nutritional values, providing 100% of the recommended daily allowance of vitamin A for 3–4‐year‐old children, 55.41% for 9–13‐year olds, and 36.94% for adults. Thus, the study demonstrated the successful preparation of fish products (powder and chapatti) blended with OFSP flour. These products are less perishable and highly enriched with β‐carotene and carbohydrates. Hence, it is an excellent alternative food source for addressing vitamin A deficiencies in both children and adults.

## AUTHOR CONTRIBUTIONS

**Habtamu Birhanu:** Data curation (equal); formal analysis (equal); investigation (equal); methodology (equal); software (equal). **Tewodros Birhanu Aycheluhim:** Conceptualization (equal); methodology (equal); project administration (equal); supervision (equal); writing – original draft (equal); writing – review and editing (equal).

## FUNDING INFORMATION

The authors declare that no funding or grant was obtained for the study.

## CONFLICT OF INTEREST STATEMENT

The authors declared that there is no conflict of interest.

## Data Availability

Additional information including raw data set supporting the study is available from the corresponding author upon reasonable request.
